# VEGF_121_b, a new member of the VEGF_*xxx*_b family of VEGF-A splice isoforms, inhibits neovascularisation and tumour growth *in vivo*

**DOI:** 10.1038/sj.bjc.6605249

**Published:** 2009-08-25

**Authors:** E S Rennel, A H R Varey, A J Churchill, E R Wheatley, L Stewart, S Mather, D O Bates, S J Harper

**Affiliations:** 1 Microvascular Research Laboratories, Department of Physiology and Pharmacology, School of Veterinary Science, University of Bristol, Southwell Street, Bristol BS2 8EJ, UK; 2 Bristol Eye Hospital, Lower Maudlin St, Bristol BS1, UK; 3 Cancer Research Technologies, Cruciform Building, Gower Street, London, UK; 4 Nuclear Medicine Research Laboratory, Bart's and the London School of Medicine, Queen Mary, London, UK

**Keywords:** VEGF, VEGF_*xxx*_b, anti-angiogenesis, splicing

## Abstract

**Background:**

The key mediator of new vessel formation in cancer and other diseases is VEGF-A. VEGF-A exists as alternatively spliced isoforms - the pro-angiogenic VEGF_xxx_ family generated by exon 8 proximal splicing, and a sister family, termed VEGF_xxx_b, exemplified by VEGF_165_b, generated by distal splicing of exon 8. However, it is unknown whether this anti-angiogenic property of VEGF_165_b is a general property of the VEGF_xxx_b family of isoforms.

**Methods:**

The mRNA and protein expression of VEGF_121_b was studied in human tissue. The effect of VEGF_121_b was analysed by saturation binding to VEGF receptors, endothelial migration, apoptosis, xenograft tumour growth, pre-retinal neovascularisation and imaging of biodistribution in tumour-bearing mice with radioactive VEGF_121_b.

**Results:**

The existence of VEGF_121_b was confirmed in normal human tissues. VEGF_121_b binds both VEGF receptors with similar affinity as other VEGF isoforms, but inhibits endothelial cell migration and is cytoprotective to endothelial cells through VEGFR-2 activation. Administration of VEGF_121_b normalised retinal vasculature by reducing both angiogenesis and ischaemia. VEGF_121_b reduced the growth of xenografted human colon tumours in association with reduced microvascular density, and an intravenous bolus of VEGF_121_b is taken up into colon tumour xenografts.

**Conclusion:**

Here we identify a second member of the family, VEGF_121_b, with similar properties to those of VEGF_165_b, and underline the importance of the six amino acids of exon 8b in the anti-angiogenic activity of the VEGF_xxx_b isoforms.

The advent of anti-neovascular therapies in cancer and eye disease has prompted a burgeoning interest in the mechanisms behind the initiation, development and refinement of new vessel formation. The growth of new vessels from those already established is termed angiogenesis and although a complex process, involving over 50 growth factors, cytokines, receptors and enzymes, is dominated, in most situations, by the 38 kDa cysteine-knot protein vascular endothelial growth factor (VEGF). Following the initial identification of VEGF (Senger *et al*, 1983) many splice isoforms were subsequently identified, eventually forming a family of mature peptides – the VEGF_*xxx*_ family (see Figure 1) (Houck *et al*, 1991). The individual family members have different expression patterns (e.g., VEGF_183_ expression is mostly restricted to the eye (Jingjing *et al*, 1999), VEGF_145_ in the reproductive system (Charnock-Jones *et al*, 1993)); unique characteristics with respect to heparin and receptor-binding properties (resulting from inclusion/exclusion of particular exon segments) and are named by amino acid size. Despite this heterogeneity they are all considered pro-angiogenic, pro-permeability vasodilators. The evidence for these properties for individual splice isoform recombinant proteins *in vivo*, however, varies from conclusive (VEGF_165_) (Leung *et al*, 1989), through mixed (VEGF_121_) (Keyt *et al*, 1996; Zhang *et al*, 2000) and scanty (VEGF_145_) (Poltorak *et al*, 1997) to absent (VEGF_206_).

In addition, it has now become evident that even the presence of six splice isoforms is an over-simplification. The pro-angiogenic potential of conventional VEGF isoforms is thought to result from the C-terminus (Keyt *et al*, 1996), more specifically the six amino acids of exon 8a – CDKPRR (Cebe Suarez *et al*, 2006; Cebe-Suarez *et al*, 2008). A sister family of splice isoforms has now been identified (see Figure 1) in which the pro-angiogenic domain (exon 8a) is replaced by the six amino acids coded for by exon 8b – SLTRKD. These exon 8b isoforms (VEGF_*xxx*_b) are exemplified by the only isoform so far extensively investigated, VEGF_165_b. VEGF_165_b has radically different properties to that of the widely studied VEGF_165_. We, and others have shown that VEGF_165_b is not pro-angiogenic *in vivo* (Woolard *et al*, 2004) and specifically and actively inhibits VEGF_165_-mediated endothelial cell proliferation and migration *in vitro* (Bates *et al*, 2002; Rennel *et al*, 2008a, 2008b), vasodilatation *ex vivo* (Bates *et al*, 2002) and *in vivo* angiogenesis assays such as rabbit corneal eye pocket, chicken chorioallantoic membrane, mesenteric and Matrigel implants (Woolard *et al*, 2004; Cebe Suarez *et al*, 2006; Kawamura *et al*, 2008). Furthermore, VEGF_165_b not only inhibits physiological angiogenesis in developing mammary tissue in transgenic animals over-expressing VEGF_165_b (Qiu *et al*, 2008) but also pathological angiogenesis in models of the proliferative ocular lesions of diabetes (retinopathy of pre-maturity) (Konopatskaya *et al*, 2006) and age-related macular degeneration (laser-induced choroidal neovascularisation, Hua *et al*, unpublished) and in six different tumour models *in vivo* (Woolard *et al*, 2004; Varey *et al*, 2008; Rennel *et al*, 2008a, 2008b). The above anti-angiogenic properties occur when delivered as VEGF_165_b-transfected cells (Woolard *et al*, 2004; Varey *et al*, 2008; Rennel *et al*, 2008a), VEGF_165_b-adenoviral constructs (Woolard *et al*, 2004) or recombinant human protein (Konopatskaya *et al*, 2006; Rennel *et al*, 2008b).

Although a significant amount of evidence on the expression and function of the VEGF_165_b isoform has already been published, there is very little evidence on other VEGF_*xxx*_b family members, and their existence and activity is still uncertain. This study, therefore, investigates the properties of a second member of the VEGF_*xxx*_b family – the peptide VEGF_121_b, addressing the hypothesis that VEGF_121_b will also show anti-angiogenic properties. We present descriptive expression data and functional data on cell migration, receptor binding and *in vivo* tumour and ocular angiogenesis *in vivo*.

## Materials and methods

### Tissue samples

Paired colon samples were from partial colon resection for carcinoma. Samples were obtained by taking biopsies of the fresh specimen from a non-necrotic central portion of the tumour and from a peripheral part of the macroscopically normal colon (*n*=15). The mean patient age was 71.5 (58–80) years, with 62% males and Duke's staging as follows: 6.7% A, 46.7% B and 46.7% C. Informed consent for obtaining the tissue was gained in compliance with local ethics committee approval.

### PCR on cDNA from human total RNA

Total RNA was extracted from human tissue using TRIZOL (Invitrogen, Carlsbad, CA, USA) (Chomczynski, 1993) and treated with DNase (Promega, Madison, WI, USA). Complementary DNA was made using oligo dT primers or 3′UTR primer (GCTCTAGAAGCAGGTGAGAGTAAGCG) as described earlier (Bates *et al*, 2002). PCR was used to identify presence of VEGF_121_b, VEGF_165_ and VEGF_165_b using specific primers; VEGF_165_/VEGF_165_b exon 7 GGCAGCTTGAGTTAAACGAACG, 3′UTR ATGGATCCGTATCAGTCTTTCCTGG, VEGF_121_b exon 5/8b GAAAAATCTCTCACCAGGAAA, 3′UTR II CTGGATTAAGGACTGTTCTG, VEGF_121_ exon 5/8a GAAAAATGTGACAAGCCGAG 3′UTR II CTGGATTAAGGACTGTTCTG, GAPDH forward TGATGACATCAAGAAGGTGGTGAAG, reverse TCCTTGGAGGCCATGTGGGCCAT and PCR products were separated and visualised using 4% agarose/ethidium bromide gel. Putative VEGF_121_b PCR products were purified using QIAquick gel extraction (Qiagen, Crawley, UK) and inserted into pGEM vector and confirmed by sequencing of plasmid.

### Production of recombinant protein

Recombinant human VEGF_121_b (VEGF_121_b) and VEGF_165_b were produced in Chinese hamster ovary cells as glycosylated dimers by Cancer Research Technologies (London, UK) as described earlier (Rennel *et al*, 2008b). VEGF_165_ and VEGF_121_ were from R&D Systems, Minneapolis, MN, USA.

### *In vitro* assays with human umbilical vein endothelial cells

Human umbilical vein endothelial cells (HUVEC) were extracted from umbilical cords from caesarean sections (St Michael's Hospital, Bristol, UK). HUVEC migration was performed as described earlier (Rennel *et al*, 2008b). Briefly, HUVECs were serum starved for 6 h and 100 000 serum-starved cells were plated into collagen-coated 8 *μ*m inserts (Millipore, Billerica, MA, USA) placed in 24-well plates with 500 *μ*l of chemoattractant in M200 medium with 0.1% v/v FCS and 0.2% w/v BSA and incubated overnight at 37°C to allow for migration. Migrating cells were stained in Mayer's haematoxylin. Migrating cells were counted (10 random fields per insert) and expressed as percentage of migrating cells in relation to the total number of seeded cells. Experiments were performed in triplicates. A peptide (Ac-SLTRKD) was generated and increasing concentrations (1 nM–100 *μ*M) peptide was combined with or without 1 nM VEGF_165_ to analyse impact on HUVEC migration.

Cell cytotoxicity was performed using a lactate dehydrogenase assay (Promega); 13 000 cells were seeded in cell culture 96-well plates in triplicates or quadruplicates in 100 *μ*l of full growth media (FGM) for 24 h. Recombinant VEGF protein was added in 100 *μ*l media with 0.1% FCS for 48 h. Conditioned media and lysed cells were spun at 250 **g** for 15 min at 4°C followed by addition of kit components, incubated for 30 min and read at 490 nm. Receptor and kinase inhibitors were added at indicated concentrations with or without 1.4 nM VEGF_121_b.

For direct counting, HUVECs were seeded in 24-well plates in triplicates in FGM 24 h followed by 48 h exposure to 1.4 nM growth factors or FGM. Cells were trypsinised and counted using a haemocytometer.

### *In vivo* tumour model

LS174t human colon carcinoma cell lines (ECACC, Salisbury, UK) (Yuan *et al*, 1996; Lee *et al*, 2000) were grown in Minimum Essential Eagle’s Medium supplemented with 10% v/v FCS, 2 mM L-glutamine and 1% non-essential amino acids (all from Sigma Aldrich, Gillingham, UK). Cells were transfected with 1 *μ*g/well of pcDNA_3_ empty vector or pcDNA_3_-VEGF_121_b, using lipofectamine plus (Invitrogen) as per manufacturer's instructions and selected using 500 *μ*g ml^−1^ geneticin (Sigma Aldrich). Conditioned media and cell lysate were analysed by western blotting to confirm expression levels of VEGF isoforms. Two million cells were injected subcutaneously into the lumbar region of nude mice (six per group). Xenotransplanted tumours were measured by calliper every 2–3 days and tumour volume was calculated according to (length × width × [length+width]/2). Mice were culled by cervical dislocation and tumours were removed when the first tumour reached 16 mm in any direction all mice were killed in that group. Injections, measurements and analysis were all carried out with the investigators blinded to group.

Tumour vessel density was counted in 10 random fields in haematoxylin and eosin stained 6 *μ*m sections frozen sections from tumours. Vessel presence was confirmed by staining by blocking in 5% v/v goat Ig for 30 min, 20 *μ*g ml^−1^ biotinylated isolectin B4 (L-2140 Sigma-Aldrich) or 1 *μ*g ml^−1^ Flk-1 (sc-6251, Santa Cruz Biotechnology, Santa Cruz, CA, USA) overnight, 2 *μ*g ml^−1^ anti-mouse biotin antibody (Vector Laboratories, Burlingame, CA, USA) for 1 h followed by avidin biotinylated enzyme complex (Vector Laboratories) for 30 min followed by DAB substrate (Vector Laboratories). Sections were examined using a Nikon Eclipse E400 microscope and photos were captured using Nikon Eclipse Net software. To visualise apoptosis, tumour sections were stained with cleaved caspase 3 antibody (1 : 400 dilution, CatNo9661 Cell Signaling Technology, Danvers, MA, USA) and positive staining areas were circled and compared to total tumour area using Image J software.

### Cell proliferation by flow cytometry analysis and direct counting

Trypsinised LS174t cells were fixed in 70% ethanol for 30 min at 4 °C followed by 100 *μ*g ml^−1^ ribonuclease (Sigma Aldrich) for 5 min. Propidium iodine (PI, 50 *μ*g ml^−1^) was added to the cells and analysed by flow cytometer.

For healthy, apoptotic and necrotic cell determination a PI-annexin V-FITC kit was used (Bender MedSystems, Vienna, Austria). Cell culture media was centrifuged at 1500 r.p.m. to collect non-adherent cells, added to trypsined cells and diluted in binding buffer to approximately 300 000 cells/ml. Cells were stained with 25 *μ*l annexin V-FITC for 10 min, washed, stained with 20 *μ*g ml^−1^ PI and analysed by flow cytometer.

For direct counting, 135 000 LS174t cells/24 well was seeded out in triplicates in media supplemented with 0.1% FCS in the presence or absence of 1 nM VEGF_121_b. Cells were trypsined and counted using a haemocytometer after 24 and 48 h.

### *In vivo* imaging of ^125^I-VEGF_165_b biodistribution

Nude mice were injected with LS174t tumours on the right hindleg. ^125^I-VEGF_121_b was generated using Iodogen tubes (Pierce Biotechnology, Cramlington, UK) and purified with NAP-10 columns (GE Healthcare, Chalfont St Giles, UK). Analysis by thin layer chromatography showed >95% purity (^125^I-VEGF_121_b/total ^125^I). Approximalty 3.2 MBq (70 *μ*g protein) was injected into the tail vein when tumours were >10 mm in diameter. Anaesthesia was maintained by 2% halothane during X-ray and scanning (NanoSPECT/CT, Bioscan, Washington, DC, USA), after 40, 70, 120, 240 or 1440 min. For biodistribution studies, 0.10 MBq (3 *μ*g) ^125^I-VEGF_121_b was injected into the tail vein and mice were culled at 120 or 240 min. Organs and tissues of interest were excised and assessed using a gamma counter (LKB Wallac 1282 Compugamma CS, Wallac, Perkin Elmer, MA, USA). Uptake was expressed as % injected dose/g tissue.

### *In vitro* saturation binding of ^125^I-VEGF_*xxx*_b to Fc-VEGFR-1

One *μ*g ml^−1^ human VEGFR-1-Fc chimaera or VEGFR-2-Fc (R&D Systems) was bound to Immulon II HB Flat 96-well plates (Thermo Labsystems, Basingstoke, UK) overnight followed by blocking with 3% w/v BSA for 2 h and binding of ^125^I-rhVEGF_121_b or ^125^I-rhVEGF_165_b for 4 h in 1% w/v BSA in triplicates. Plates were washed three times with PBS+0.05% v/v Tween in between each step. To detach the complex 10% w/v SDS was added and counted in a gamma counter. Data were calculated as c.p.m. and expressed as % binding compared with maximum binding concentration.

### SDS–PAGE and immunoblotting of tissue and cell lysate

Tissue lysates from transfected cells or human colon tissue were extracted using RIPA buffer (50 mM Tris, 150 mM NaCl, 1% v/v NP-40, 0.25% w/v Na-deoxycholate, 1 mM EDTA, 1 mM phenylmethylsulfonyl fluoride, 1 *μ*g ml^−1^ of each of aprotinin, pepstatin and leupeptin) and homogenised using a polytron and spun at 12 000 r.p.m. for 10 min at 4°C. Lysates were separated on 15% SDS–PAGE, transferred to polyvinylidene fluoride membranes, blocked in 10% w/v dry milk, incubated with a mouse monoclonal anti-human VEGF_*xxx*_b antibody (2 *μ*g ml^−1^, R&D Systems) for 1 h followed by horse radish peroxidase conjugated anti-mouse antibody (1/5000, Pierce Biotechnology) for 1 h. Membranes were developed using supersensitive West Femto Maximum Sensitive Substrate (Pierce Biotechnology). Densitometry analysis of western blots was performed using Image J. VEGF_121_b is detected by immunoblotting using anti-VEGF antibodies (A-20 Santa Cruz or AF-293 R&D Systems) and the commercial VEGF_*xxx*_b antibody (A56/1, R&D Systems, cat no. MAB3045). Commercial ELISA for VEGF_165_b (R&D Systems) and panVEGF (Duoset R&D Systems) can pick up VEGF_121_b but panVEGF ELISA underestimates the VEGF_121_b levels (and VEGF_165_b) by approximately 40% (Varey *et al*, 2008).

### Oxygen-induced retinopathy mouse model

The oxygen-induced retinopathy (OIR) model was performed as described earlier (Smith *et al*, 1994; Konopatskaya *et al*, 2006) with minor modifications. Neonatal C57/Bl6 mice and nursing CD1 dams were exposed to 75% oxygen between P7 and P12. Return to room air induced hypoxia in the ischaemic areas. On P13, mice received either VEGF_121_b (1 or 10 ng) or Hank's buffered solution in 1 *μ*l intraocular injections using a Nanofil syringe fitted with a 35 gauge needle (WPI, Sarasota, FL, USA) into the left eye under isoflurane anaesthesia. On P17, both eyes were dissected, fixed in 4% paraformaldehyde for 4 h at 4°C and retinas were dissected. Retinas were permeabilised in PBS containing 0.5% Triton X-100 and 1% bovine serum albumin, stained with 20 *μ*g ml^−1^ biotinylated isolectin B4 (Sigma Aldrich) in PBS pH 6.8, 1% Triton-X100, 0.1 mM CaCl_2_, 0.1 mM MgCl_2,_ followed by 20 *μ*g ml^−1^ ALEXA 488-streptavidin (Molecular Probes, Eugene, OR, USA) and flat mounted in Vectashield (Vector Laboratories). Quantification of neovascular and ischaemic areas were performed in a blinded fashion using Photoshop CS3 along with Image J and expressed as percentage of total retinal area (=normal+ischaemic+neovascular).

### Statistical analysis

Statistical analysis was performed using GraphPad Prism software (version 4.0cx). Data are given as means±s.e.m. or means±95 % confidence interval when stated. One-way ANOVA was used to compare migration, cytoprotection, tumour growth and retinal vascularisation. *T*-test was used to compare tumour growth, apoptosis and microvascular density and paired *t*-test used for retinal areas.

## Results

### Expression of VEGF_121_b in human colon tissue and its reduction in colon carcinoma

**Figure 2:**
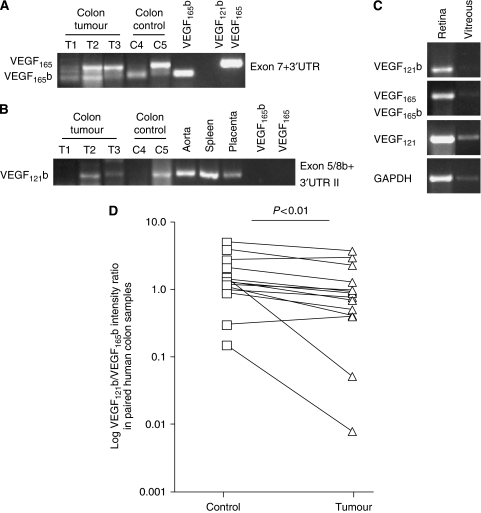
VEGF_121_b is expressed in human tissue and the expression is reduced in human colon tumours. (**A–C**) PCR on cDNA from different human tissues using primers to detect VEGF_121_b, VEGF_165_b and VEGF_165_. VEGF_165_ and VEGF_165_b are detected in colon cDNA both in tumour and adjacent control colon (**A**). VEGF_121_b is detected in colon tissue, aorta, spleen and placenta using VEGF_121_b-specific primers (**B**). VEGF_121_, _165_, _121_b and _165_b can be found in the retina and to a lesser extent in the human vitreous (**C**). (**D**) Densitometry analysis of VEGF_121_b and VEGF_165_b protein expression in matched human control colon samples and colon tumours analysed by western blot using a VEGF_*xxx*_b-specific antibody (*n*=14 pairs, *P*=0.0067 paired *t*-test).

Earlier, we reported a shift in expression of VEGF_*xxx*_b isoforms in human malignant melanoma (Pritchard-Jones *et al*, 2007), prostate (Woolard *et al*, 2004; Rennel *et al*, 2008a) and colon carcinoma (Varey *et al*, 2008) leading to less anti-angiogenic VEGF_*xxx*_b splice isoforms in cancer tissue. The ELISA used to quantify the levels of VEGF_*xxx*_b isoforms does not distinguish between the individual members of the VEGF_*xxx*_b isoform family. Earlier RT–PCR used to detect VEGF_*xxx*_b isoforms (using exon 7 and 3′UTR primers) did not detect VEGF_121_b (see Figure 2A) as the forward primer was in exon 7 (spliced out in VEGF_121_b and VEGF_121_). To identify VEGF_121_b, mRNA primers were used that spanned the exon 5/exon 8b boundary. These primers detected VEGF_121_b but not VEGF_165_b or VEGF_165_ (see Figure 2B). VEGF_121_b was detected in normal tissue such as aorta, spleen, placenta and some normal and tumour colon tissue (see Figure 2B), which also expressed VEGF_165_ and VEGF_165_b (see Figure 2A) and retina and vitreous (Figure 2C). Presence of VEGF_121_b was confirmed by sequencing of PCR products.

Measurement of VEGF levels in an earlier study on human colon carcinoma showed that the angiogenic VEGF isoforms were upregulated compared with matched colon tissue whereas the levels of anti-angiogenic VEGF_*xxx*_b isoforms remained unchanged. Western blot on that tissue showed two bands consistent with expression of VEGF_121_b and VEGF_165_b (Varey *et al*, 2008). Quantification of the western blot analysis of paired human colon tissues from tumour and adjacent normal colon tissue indicates a downregulation of VEGF_121_b in tumour tissue compared to VEGF_165_b (see Figure 2D, *P*<0.01 paired *t*-test, *n*=14 pairs).

### VEGF_121_b is secreted, inhibits migration and reduces apoptosis in endothelial cells

**Figure 3:**
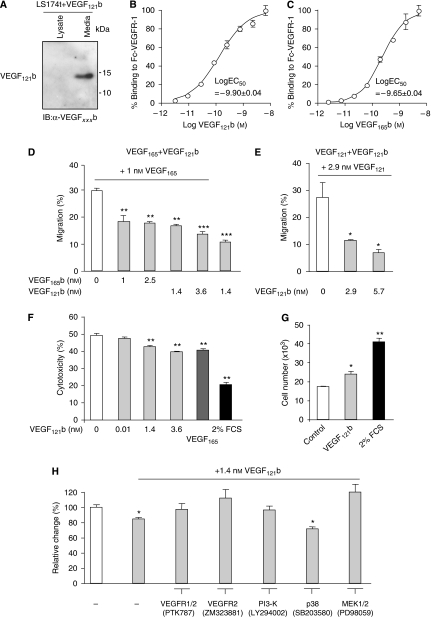
VEGF_121_b inhibits migration of endothelial cells and inhibits serum starved induced cell death in endothelial cells through classical VEGFR-induced pathways. (**A**) VEGF_121_b is found in the media on transfected cancer cells. (**B**–**C**) VEGF_121_b has similar saturation binding kinetics to VEGFR-1 as VEGF_165_b. (**D**–**E**) VEGF_121_b inhibits VEGF_165_ (**D**) or VEGF_121_ (**E**) induced migration of HUVEC. Cells were allowed to migrate through 8 *μ*m pore inserts with increasing concentrations of VEGF_121_b (0–6 nM) or VEGF_165_b (0–2.5 nM) in the presence of 1 nM VEGF_165_ or 2.9 nM VEGF_121_. Data plotted as percentage migration compared to total number of seeded cells (**F**) VEGF_121_b is cytoprotective to a similar degree as VEGF_165_ in HUVEC. Release of lactate dehydrogenase as a measurement of cell viability was monitored after 48 h of serum starvation in the presence of increasing amounts of VEGF_121_b (0–3.6 nM), 1 nM VEGF_165_ or full growth media (FGM) with serum and growth factors. (**G**) Similar cytoprotection of VEGF_121_b was observed in serum starved HUVEC as cell number was not reduced by the same amount after 48 h of cell starvation. (**H**) VEGF_121_b cytoprotection is mediated via VEGFR-1 and -2, P13-K, MEK1/2. Inhibition of VEGFR1/2=200 nM PTK787, VEGFR-2=10 nM ZM323881, P13- K=15 *μ*M LY294002, p38=10 *μ*M SB203580 and MEK1/2=15 *μ*M PD98059. (One-way ANOVA, Dunnett's *post hoc* test, 0 nM or control *vs* addition, ^*^P<0.05, ^**^P<0.01, ^***^P<0.001).

To determine whether VEGF_121_b can be secreted by colon cells, VEGF_121_b recombinant cDNA was transfected into colon carcinoma cells. Western blotting of the lysate and the media showed that VEGF_121_b was found pre-dominantly in the media, indicating that it is freely secreted (see Figure 3A), in contrast to cells that were not transfected with the VEGF_121_b plasmid, in which no VEGF_121_b was seen in the media or the lysate. VEGF_121_b binding to VEGFR-1 was analysed by incubating increasing concentrations of radiolabelled VEGF_121_b with immobilised VEGFR-1 (see Figure 3B). The saturation-binding kinetics of VEGF_121_b (see Figure 3B, log_10_EC_50_=−9.9±0.04) was similar to that for VEGF_165_b measured at the same time (EC_50_=−9.65±0.04, Figure 3C).

We have shown earlier that VEGF_165_b conditioned media (Bates *et al*, 2002; Rennel *et al*, 2008a) or recombinant human VEGF_165_b (Woolard *et al*, 2004; Rennel *et al*, 2008b) inhibits VEGF_165_-induced migration of endothelial cells. Migration of HUVEC was inhibited by the addition of VEGF_121_b either when stimulated with VEGF_165_ (see Figure 3D, VEGF_165_ *vs* any treatment *P*<0.05) or VEGF_121_ (see Figure 3E, VEGF_121_ *vs* any treatment *P*<0.05). The level of inhibition was similar to that observed with VEGF_165_b (see Figure 3D) and no additional significant reduction in migration was observed with the combination of VEGF_121_b and VEGF_165_b (see Figure 3D). Addition of up to 100 *μ*M peptide of the last six amino acids had no effect on VEGF_165_-induced HUVEC migration (data not shown) indicating that the 3D structure and or the full length of the VEGF_*xxx*_b protein is needed for inhibition.

VEGF_165_ is known to protect endothelial cells from apoptosis and incubation of HUVECs with recombinant VEGF_121_b was equally cytoprotective (see Figure 3F, no addition *vs* any treatment *P*<0.01). This resulted in a small increase in cell number induced by treatment with VEGF_121_b (see Figure 3G). To determine the mechanism of this cytoprotective effect of VEGF_121_b, HUVECs were treated with inhibitors of receptor tyrosine kinases and signalling kinases (see Figure 3H). Inhibition of VEGFR-1 and VEGFR-2 by 100 nM PTK787 resulted in an inhibition of the cytoprotective effect of VEGF_121_b, as did incubation with the VEGFR-2-specific antagonist ZM323881 (10 nM). The cytoprotective effect was blocked by inhibitors of PI3Kinase (LY294002) and MEK1/2 (PD98059), but not by inhibitors of p38 MAPK (SB203580, see Figure 3H).

### Over-expression of VEGF_121_b reduces tumour growth through a direct effect on endothelial cells

**Figure 4:**
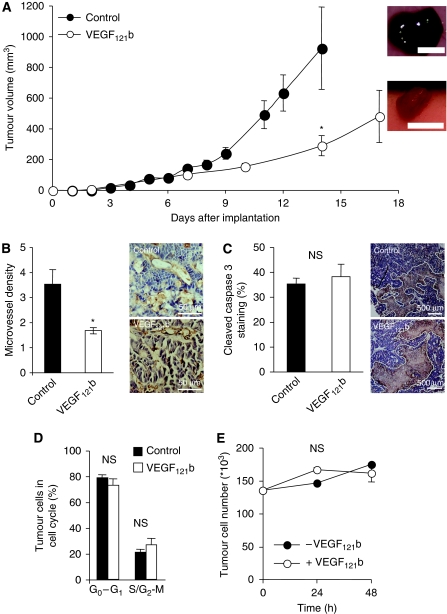
VEGF_121_b reduces tumour growth in nude mice bearing colon carcinoma tumours by reducing tumour vessel ingrowth. (**A**) LS174t human colon carcinoma cells were transfected with pcDNA_3_-VEGF_121_b or empty pcDNA3 plasmid and injected subcutaneously into nude mice and tumour growth was monitored over time. Over-expression of VEGF_121_b resulted in a reduced tumour growth and contained less blood compared with control cells (inserted images). Scale bar=10 mm. (**B**) Immunohistochemistry staining of tumour sections for VEGFR-2 visualise microvessels (inserted images). Quantification of microvascular density showed significantly fewer blood vessels per unit area than control tumours. Each point represents the mean of 10 random analysed fields and six tumours per treatment were examined (^*^P<0.05 unpaired *t*-test). (**C**) Cleaved caspase 3 staining of tumour sections (inserted images) showed no significant difference in apoptosis. (**D**, **E**) VEGF_121_b has no effect on LS174t colon carcinoma growth *in vitro*. Over-expression of VEGF_121_b in LS174t colon carcinoma cells had no effect on cell proliferation analysed by FACS (**D**). Addition of 1 nM VEGF_121_b had no effect of cell number analysed by direct counting of cells over 48 h (**E**).

We have shown earlier that over-expression of VEGF_165_b reduces tumour growth in five different tumour types (Woolard *et al*, 2004; Varey *et al*, 2008; Rennel *et al*, 2008a), but VEGF_165_b does not directly affect proliferation or apoptosis of LS174t cells (Varey *et al*, 2008). To verify whether VEGF_121_b could inhibit tumour growth as well, LS174t colon carcinoma tumour cells were transfected with plasmid containing VEGF_121_b or cloning vector alone (control). Expression levels were confirmed by immunoblot on lysate and conditioned media showing that VEGF_121_b was secreted and found in the media (see Figure 3A). Cells were injected into the back of nude mice and tumour growth was monitored over time. VEGF_121_b reduced tumour growth compared with empty vector (290±66 mm^3^ *vs* 925±268 mm^3^ after 14 days, *P*<0.05, unpaired *t*-test Welch correction, see Figure 4A). VEGF_121_b tumours were smaller and excised tumours were less haemorrhagic than those with empty vector (see Figure 4A inserted images). Immunohistochemical staining was performed to visualise blood vessel distribution (see Figure 4B for representative VEGFR-2 staining). Analysis of the vessel density in 10 random fields in each tumour showed a decreased number of vessels in VEGF_121_b tumours (control *vs* VEGF_121_b, 3.5±0.6 *vs* 1.7±0.1, *P*<0.05 unpaired *t*-test *n*=6 tumours per treatment 10 fields analysed per tumour, Figure 4B). VEGFR-2 staining of tumour sections showed expression of VEGFR-2 in the tumour vessels and not in the general tumour mass (see Figure 4B inserted images) indicating an effect of VEGF_121_b on the endothelium rather than an anti-proliferative effect on the tumour cells. Earlier analysis has shown absence of VEGFR-1 and VEGFR-2 expression in LS174t cells (Rennel *et al*, 2008b). To determine whether VEGF_121_b expression induced apoptosis in the tumour cells, tumours were stained for cleaved caspase 3. There was no change in caspase staining in control *vs* VEGF_121_b-expressing tumours (see Figure 4C).

Transfected LS174t colon cells were analysed by flow cytometry and VEGF_121_b had no effect on proliferation (see Figure 4D control *vs* VEGF_121_b, S/G_2_-M 21±2.3 *vs* 27±4.4, *P*=0.36 unpaired *t*-test). In addition, addition of recombinant VEGF_121_b had no effect on tumour cell number (see Figure 4F). These data indicate that VEGF_121_b reduced tumour growth by reducing the tumour-driven angiogenesis, rather than a direct effect on the tumour cells themselves.

### Biodistribution of intravenous VEGF_121_b

**Figure 5:**
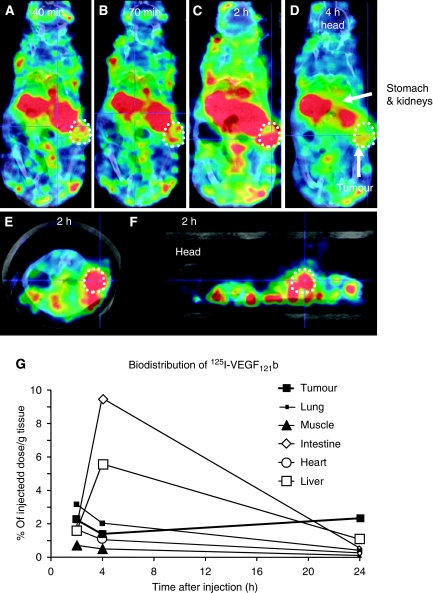
*In vivo* distribution of ^125^I-VEGF_121_b in tumour-bearing mice. Tumour-bearing mice received an intravenous injection of ^125^I-VEGF_121_b and 3D imaged using NanoSPECT/CT. (**A**–**D**) Time course for biodistribution of ^125^I-VEGF_121_b after tail vein injection using transverse sections. (**E**) Coronal. (**F**) Para-sagittal through the centre of the tumour. The tumour is circled and arrows indicate different organs. (**G**) Quantification uptake into different organs and tissues over time. Data expressed as % in tissue relative to the total injected dose, per gram of tissue.

The results above indicate that VEGF_121_b could be a potential therapeutic agent in angiogenic diseases, if the uptake into neovascular tissues was sufficient. To determine how intravenously (i.v.) injected recombinant VEGF_121_b is distributed *in vivo*, ^125^I-VEGF_121_b was injected i.v. into tumour-bearing mice and imaged using high-resolution single photon emission computed tomography (NanoSPECT/CT). Radiolabelled VEGF_121_b was distributed quickly through the mouse and images acquired at 40 min post injection (p.i.) showed accumulation in organs of metabolism and secretion. Thereafter, the overall signal gradually declined because of excretion/degradation of the radiolabelled protein. Figure 5A shows whole body images at different time p.i. Uptake of radioactivity can be seen in the tumour as well as abdominal tissues such as stomach and kidney. Coronal and sagittal sections also visualised uptake in the tumour at 2 h p.i. (see Figure 5E-F). Biodistribution of tissue and tumour indicated that approximately 2.5% of the total ^125^I is found in the tumour at 2 h and stays at a similar level for up to 24 h (see Figure 5G). Intestine and liver showed high uptake with a maximum at 4 h. No obvious adverse haemodynamic effects were seen in the animals.

### VEGF_121_b rescues retinal vasculature by reducing neovascularisation and ischaemia

**Figure 6:**
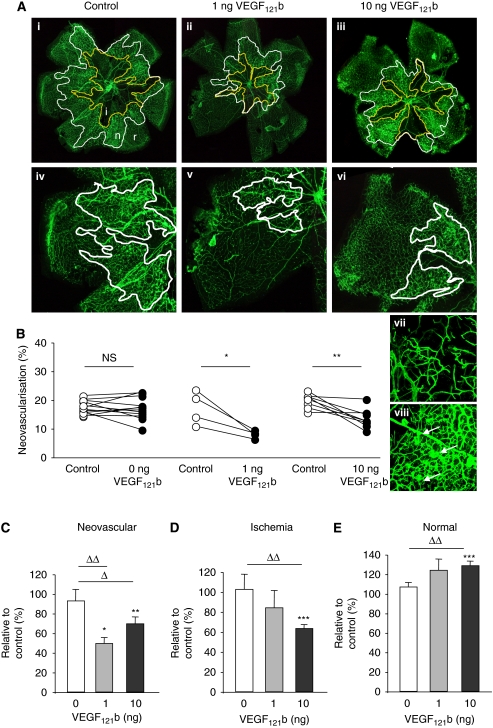
VEGF_121_b reduces hypoxia-induced retinal neovascularisation after single intraocular injection. The oxygen-induced retinopathy mouse model leads to neovascularisation induced by hypoxia. (**A**) Flat mounted mouse retinas with vessels visualised by isolectin staining. Ischaemic and neovascularised areas are circled (i, ischaemic, n, neovascular and r, normal retinal vessels). Normal retinal vessels (vii) are slender compared to neovascular vessels (viii). (**B**) Quantification of neovascularisation, ischaemic and normal vessel growth compared to matched uninjected control eye (paired *t*-test, ^*^P<0.05, ^**^P<0.01). **(C**–**E**). Distribution of ischaemic, neovascular and normal vessel growth in injected retinas relative to uninjected eye showed that VEGF_121_b reduced neovascularisation (**C**) and the ischaemic areas (**D**) leading to an increase in the revascularisation (**E**). (One-way ANOVA, Bonferroni *post hoc* test, Δ*P*<0.05, ΔΔ*P*<0.01 compared to vehicle injected=0 ng).

We have shown earlier that the anti-angiogenic VEGF_165_b can inhibit retinal neovascularisation in an OIR mouse model when administered as a single intraocular injection (Konopatskaya *et al*, 2006). In this model retinal vaso-obliteration occurs in the central retina when pups are exposed to high oxygen (from P7 to P12) and when returned to room air the relative hypoxia induces neovascularisation with a peak reached at P17. Retinas were flat mounted and stained with isolectin to visualise vessels with neovascularisation occurring at the edge of the central ischaemic area (see Figure 6A). Blind quantification of the injected and uninjected contralateral retina, showed reduced neovascularisation after 1 or 10 ng VEGF_121_b (see Figure 6B-C, uninjected control *vs* 1 or 10 ng VEGF_121_b *P*<0.05 or control injection *vs* 1 or 10 ng VEGF_121_b *P*<0.05). Injection of 10 ng VEGF_121_b reduced the ischaemic area, and increased the area of normal vasculature (see Figure 6D-E, control injection *vs* 10 ng VEGF_121_b *P*<0.05 for ischaemic and normal vessel growth). The overall result was an increase in the proportion of normal vasculature in these retinae (see Figure 6E). Injection of control solution had no effect compared with contralateral eye (see Figure 6B uninjected control *vs* control injected eye, *P*>0.05, paired *t*-test).

## Discussion

The existence of a second family of VEGF isoforms with overtly different properties from that of the conventional VEGF species required a radical re-evaluation of VEGF biology, not only academically – thousands of manuscripts have attempted to quantify ‘VEGF’ in body fluids or tissues using primers, probes or antibodies that would not distinguish these contrasting families; but also in terms of the pathogenesis of disease and the potential clinical importance of VEGF_*xxx*_/VEGF_*xxx*_b balance, because these families derive from the same gene. VEGF_165_b is widely expressed (Bates *et al*, 2002, 2006; Cui *et al*, 2004; Perrin *et al*, 2005; Pritchard-Jones *et al*, 2007; Varey *et al*, 2008) and may form the pre-dominant isoform in many tissues (Perrin *et al*, 2005; Varey *et al*, 2008). The physiological role of these isoforms is only just now being elucidated in normal tissues, and it seems that they act as a brake on excess angiogenesis during conditions of controlled growth or VEGF_*xxx*_ expression. Examples include development of the ovary, where the VEGF_*xxx*_b levels are maintained to prevent angiogenesis into the developing germ bud and so prevent formation of a testicular artery (VEGF_*xxx*_b antibodies are angiogenic in this model, Artac *et al*, in press), in the virgin mammary fat pad, where VEGF_*xxx*_ levels are prevented from inducing angiogenesis until lacteal formation, when VEGF_*xxx*_b levels fall (and prevention of this fall results in reduced milk production) (Qiu *et al*, 2008), and in the eye where a relative drop in VEGF_*xxx*_b levels is accompanied with angiogenesis in the retina in diabetes (Perrin *et al*, 2005). Increased VEGF_*xxx*_b levels also seem to be required for normal pregnancy, as a failure to upregulate VEGF_*xxx*_b in first trimester pregnancies is associated with subsequent maternal–fetal pathology (Bills *et al*, 2009).

The development of an angiogenic milieu within a stable and non-angiogenic tissue, may depend to some degree on changes in gene (over) transcription or suppression but also on changes in mRNA splicing control. The control of splicing between the two VEGF isoform families has begun to be elucidated, and it has been shown that multiple stimulatory signals can differentially affect the choice of splice site in the terminal exon, including hypoxia (Varey *et al*, 2008), IGF, TNF and PDGF (Nowak *et al*, 2008), which all favour proximal splice site selection in exon 8, and hence angiogenic isoforms, and the latter three growth factors suppress distal splicing, whereas hypoxia seems not to affect it. TGF-*β*, on the other hand, seems to favour distally spliced isoforms (Nowak *et al*, 2008). This seems to be through mechanisms involving splice factors such as ASF/SF2 and SRp55. Additionally, alteration of splicing has been seen in human and animal models of disease including in Denys Drash Syndrome (Schumacher *et al*, 2007), kidney, prostate and colon cancer (Bates *et al*, 2002; Woolard *et al*, 2004; Varey *et al*, 2008; Rennel *et al*, 2008a), malignant melanoma (Pritchard-Jones *et al*, 2007) where the PSS is favoured and animal models of glaucoma where the DSS is favoured (Ergorul *et al*, 2008). However, there is very little known about regulation of splice site choice in the terminal exon from the exon 5 splice donor site, but the earlier studies on hypoxia indicate that this is unlikely to be a regulatory factor for VEGF_121_b.

The most studied VEGF_*xxx*_b family member – VEGF_165_b – has been clearly shown not to be angiogenic. Other researchers have shown that the six amino acids of exon 8b are required for the anti-angiogenic activity of VEGF_165_b because artificially generated VEGF_159_ lacking both exons 8a and b, which is not angiogenic (Cebe Suarez *et al*, 2006). Exon 8a seems to be required for heparin and neuropilin-1 binding and full activation of the VEGFR-1 and -2, as it is likely to interfere with the correct folding and 3D structure of the protein, resulting in differential activation of VEGFR-2 tyrosine phosphorylation, and hence downstream signalling (Cebe Suarez *et al*, 2006; Cebe-Suarez *et al*, 2008; Kawamura *et al*, 2008). Here, we present for the first time new data suggesting that the presence of exon 8b in a different VEGF_*xxx*_b family member endows it with similar properties to VEGF_165_b: VEGF_121_b inhibits experimental endothelial cell migration *in vitro* and new vessel formation *in vivo* in tumour and non-tumour-related angiogenesis. The VEGF_*xxx*_b family members seem to share similar properties.

We have shown that VEGF_121_b has similar bio-distribution after i.v. injection to VEGF_165_b and a similar proportion of injected bolus reaching a single implanted tumour – with around 2% for VEGF_121_b and 6% for VEGF_165_b (Rennel *et al*, 2008b). Furthermore, we have shown in this study and in our earlier work (Woolard *et al*, 2004) that these two VEGF_*xxx*_b family members bind the conventional tyrosine kinase VEGF receptors. VEGF_165_b does not bind neuropilin-1 (Kawamura *et al*, 2008), and there is evidence that VEGF_121_ also binds neuropilin poorly, suggesting that VEGF_121_b would not bind it either. von Wronski *et al* (2007) have highlighted the likelihood that neuropilin-1 binds the residues coded for by exon 8a and replacement by 8b results in VEGF isoforms that do not seem to show neuropilin-1 binding (Cebe Suarez *et al*, 2006).

Although we have shown that VEGF_165_b and VEGF_121_b bind to the conventional VEGF receptors, further work will be required to investigate the mechanistic differences between VEGF_121_b and its corresponding isoform VEGF_121_. Studies into the mechanistic contrast between VEGF_165_b and VEGF_165_ are more advanced with increasing evidence now emerging that VEGF_165_b, although binding to VEGFR-2 with equal affinity as VEGF_165_, is not simply a classical competitive inhibitor. VEGF_165_b does stimulate the phosphorylation of VEGFR-2 but qualitatively in a unique way. Although VEGF_165_b does lead to the phosphorylation of tyrosine residues of the VEGF receptor, tryptic phosphopeptide mapping and the use of phosphosite-specific antibodies have shown that VEGF_165_b is considerably poorer than VEGF_165_ in inducing phosphorylation of the angiogenic positive regulatory site Y1052 (Y1054 in human sequence) in VEGFR-2 (Kawamura *et al*, 2008). This study also confirmed that the ability of different VEGF isoforms to induce angiogenesis correlated with their abilities to bind the VEGF co-receptor neuropilin-1 (Kawamura *et al*, 2008). Whether or not VEGF_121_b activates VEGFR-2 in a similar way is yet to be investigated, as are the potential effects of simultaneous VEGF_121_b and VEGF_165_b administration of receptor activity, angiogenesis or tumour growth.

Another significant finding here is that VEGF_121_b seems to act as a survival factor for human endothelial cells. This activity seems to be VEGFR dependent, and uses classical survival pathways such as the PI3 kinase and MEK/ERK pathways. This provides a potential explanation for the surprising finding that the ischaemic region in the retinal neovascular preparations was reduced by VEGF_121_b administration. This is of particular interest as it suggests a vascular normalisation in the retina, leading to the possibility that VEGF_121_b may be of use in ischaemic retinal diseases associated with angiogenesis such as diabetic retinopathy.

In summary, we provide new information about the existence, expression and activity of a second member of the anti-angiogenic VEGF_*xxx*_b family. Our data confirm that VEGF_121_b has similar inhibitory properties on the process of *in vivo* angiogenesis and the components of angiogenesis *in vitro* as does VEGF_165_b, supporting the notion that the replacement of the terminal six amino acids with those coded for by exon 8b is of crucial importance in converting the dominant pro-angiogenic growth factor into a positively anti-angiogenic molecule that may have broad therapeutic potential. Further studies will be required to elucidate its mechanism of action of VEGF_121_b and its potential clinical value along with its other family member VEGF_165_b.

## Figures and Tables

**Figure 1:**
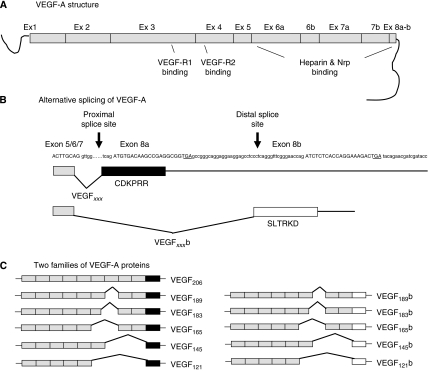
Alternative splicing of VEGF-A generating VEGF_*xxx*_ and VEGF_*xxx*_b isoforms. (**A**) VEGF-A contains eight exons with receptor binding found in exons 3 and 4, heparin and neuropilin binding in exons 6 and 7 and 8a. (**B**) Alternative splicing of the C-terminal end using proximal splice site generating VEGF_*xxx*_ isoforms or distal splice site generating VEGF_*xxx*_b isoforms. This splicing leads to an alterative last six amino acids (CDKPRR or SLTRKD). (**C**) The C-terminal splicing leads to the possibility of two sister families of VEGF-A isoforms; VEGF_*xxx*_ and VEGF_*xxx*_b, differing only in the C-terminal.
